# Statistical predictions with glmnet

**DOI:** 10.1186/s13148-019-0730-1

**Published:** 2019-08-23

**Authors:** Solveig Engebretsen, Jon Bohlin

**Affiliations:** 1 Division for Infection Control and Environmental Health, Department of Infectious Disease Epidemiology and Modelling, Norwegian Institute of Public Health, Oslo, Norway; 2 Oslo Centre for Biostatistics and Epidemiology, Department of Biostatistics, University of Oslo, Oslo, Norway; 3 Centre for Fertility and Health (CEFH), Norwegian Institute of Public Health, Oslo, Norway; 4 Faculty of Veterinary Science, Department of Production Animals, Norwegian University of Life Science, Ås, Norway

**Keywords:** Elastic net, glmnet package, Statistical prediction, Ultra-high dimensional regression

## Abstract

Elastic net type regression methods have become very popular for prediction of certain outcomes in epigenome-wide association studies (EWAS). The methods considered accept biased coefficient estimates in return for lower variance thus obtaining improved prediction accuracy. We provide guidelines on how to obtain parsimonious models with low mean squared error and include easy to follow walk-through examples for each step in R.

**Electronic supplementary material:**

The online version of this article (10.1186/s13148-019-0730-1) contains supplementary material, which is available to authorized users.

## Main text

Statistical prediction methods have recently become popular in epigenome-wide association studies (EWAS), especially for predicting epigenetic age [1–4]. Variable selection and prediction from datasets of ultra-high dimensions, such as those typically encountered in EWAS, can however be challenging due to comparatively low sample sizes (*n* << *p*, few samples *n* compared to many predictors *p*). The elastic net [5] from the “glmnet” package [6] is a generalization of several *n* << *p* shrinkage-type regression methods and includes established methods such as Lasso [7] and Ridge regression [8] as special cases. The least angle regression algorithm is used to estimate the parameters for all elastic net methods [5, 9].

### Trade-off between bias and variance

The mean squared error (MSE) is the average of the squared difference between the observations and the estimated values from the fitted model. The MSE can be decomposed into a sum of the bias and the variance, and when selecting an estimation method, there is a trade-off between these two components. The Stein theorem states that as long as the dimension of the model with parameters to be estimated simultaneously is larger than or equal to 3, biased estimators may be preferable to unbiased estimators due to lower MSE [10]. Shrinkage-based methods estimate coefficients in a biased manner and have roots that can be traced back to the James-Stein estimator [10, 11]. These methods aim at improving prediction accuracy by shrinking the estimated parameters or setting them to zero, which decreases the variance.

### Elastic net

The elastic net is an example of a shrinkage method which contains both Lasso and Ridge regression as special cases. An attractive property of the elastic net is its ability to handle *n* << *p* problems [5]. The elastic net allows tuning of the penalty term

$$ \lambda \left(\frac{1}{2}\left(1-\alpha \right){\boldsymbol{\beta}}^2+\alpha \left|\boldsymbol{\beta} \right|\right), $$

of the equation [6]:

$$ \underset{\left({\beta}_0,\boldsymbol{\beta} \right)}{\min }{\left(\boldsymbol{y}-{\beta}_0-{\boldsymbol{X}}^T\boldsymbol{\beta} \right)}^2+\lambda \left(\frac{1}{2}\left(1-\alpha \right){\boldsymbol{\beta}}^2+\alpha \left|\boldsymbol{\beta} \right|\right), $$

through the parameter *α*. The parameter *α* controls the type of shrinkage, with important consequences for the properties of the estimation method. The penalty parameter *λ* controls the amount of shrinkage. The glmnet package thus offers many different types of regression methods that can be chosen both for variable selection and feature prediction in *n* << *p* settings, depending on the problem and data at hand. Lasso (*α* = 1 in the equation above, default option in the glmnet package [6]) has an *ℓ*_1_ penalty on the parameters and performs both parameter shrinking and variable selection. The other end, *α* = 0, gives Ridge regression with a *ℓ*_2_ penalty on the parameters, which does not have the variable selection property. It can be seen from the above elastic net equation that setting the *α* parameter anywhere between the values 0 < *α* < 1 gives a penalty term dominated by the end point the *α* parameter is closest to. Lasso performs automatic variable selection and is most likely the preferred method when few sites (CpGs) are expected to be selected for prediction compared to the total number of sites in the data [6]. This is typically the norm in EWAS data with ultra-high dimensions. On the other hand, if a large fraction of CpGs are expected to be associated with a given outcome, Ridge regression (*α* = 0) should most likely be favored as no variable selection is performed [6, 12]. Setting *α* = 0.5 may be a preferred option when the fraction of CpG sites are assumed to be somewhere in between what is expected for the Lasso and the Ridge regression methods [6]. Another important drawback with Lasso is that it selects at most *n* predictors. Hence, for a dataset with a small sample size *n*, the number of CpGs selected for prediction with Lasso will never be greater than the number of samples. While no variable selection is performed using *α* = 0 (Ridge regression), a small number *ε* > 0 can be added to *α*, effectuating the *ℓ*_1_ penalty term used for variable selection [5]. Ridge regression and Lasso regression differ in how they handle correlated variables. While Ridge regression shrinks correlated variables toward each other, Lasso typically selects one. Therefore, Ridge regression tends to perform better than Lasso when the predictors are highly correlated [13]. Due to the unpredictable manner in which Lasso handles correlated predictors, a small number *ε* can be subtracted from *α* so that more correlated predictors are included in the model [6]. Furthermore, setting *α* to a value slightly below 1 allows the model to include more predictor variables than samples.

### Choosing the tuning parameters *λ* and *α*

The tuning parameters *λ* and *α* can be chosen by *k*-fold cross validation [6]. In glmnet, the default value for *k* is 10. Consider first the problem of finding the optimal *λ* from a grid of values, for a fixed *α*. The data are first split randomly into *k* equally sized blocks (folds). For each value of *λ* and for each block, the model is fitted to the data in the remaining *k* − 1 blocks. The fitted model is then used to estimate the prediction error in the block that was left out. The same procedure is repeated for all *k* blocks, resulting in an estimate of the prediction error for each *λ*. The penalty parameter *λ* can be chosen, for instance, as the minimizer of the prediction error (i.e., MSE). For a more parsimonious model, the “one standard error rule” [14] can be applied, in which the selected model is the one with the largest *λ* within one standard error of the minimum prediction error. Although “the one standard error rule” can produce a model with fewer predictors, it usually results in increased MSE and more biased parameter estimates. Cross validation can also be used to select *α*, or the elastic net method, from a grid of values, through a nested cross validation procedure. The combination of *α* and *λ* minimizing the prediction error can then be chosen. We have included a walk-through guide in R on how to estimate both *α* and *λ* with the elastic net, as well as carrying out predictions, in Additional file 1.

It may be difficult to obtain a clear understanding of the limitations and possibilities offered by shrinkage methods for prediction of *n* << *p* models due to the many implicit assumptions hidden in such methods [15]. Bias increases with the penalty parameter *λ*, as can be seen from the equation above. Given equal MSE, it is often desirable to choose the most parsimonious model (Occam’s rule) [5], as parsimonious models are often more interpretable. There could of course be reasons not to choose the most parsimonious model (e.g., Lasso’s handling of correlated predictors [6]) but then this should be justified. Nevertheless, the only way to properly validate the final selected predictor model is to assess its performance on an independent test set. We give an example in Additional file 1 of how variable selection can be performed on data from the Illumina Human Methylation 450k platform where the aim is to train a simple model for age prediction. The number of folds used for training and prediction can be adjusted according to the number of samples in the dataset. It should be noted that cross validation is performed by random selection of the *k*-folds. If the obtained results are to be duplicated at a later stage, it is recommended that a seed is specified. It is also possible to fix the penalty parameter *λ*. The smaller the penalty parameter *λ* is, the closer the elastic net coefficient estimate is to the least squares estimate, as the influence of the penalty term in the elastic net equation above will diminish. It is, however, impossible to carry out least squares estimation when the number of explanatory variables in the model exceeds the number of samples [7].

### Standard errors

Statistical testing is not directly possible using the elastic net, as no standard errors for the estimated parameters (i.e., slope coefficients) are computed directly. There is some discussion concerning the most appropriate methods to estimate variances and perform hypothesis testing for Lasso [16], but there seems to be no general agreement as of yet, not least due to Lasso’s unpredictable variable selection [17, 18]. For instance, bootstrapping is one method that can be applied for performing statistical inference on the estimated coefficients [19], but may be very time consuming on large datasets, depending on both the number of samples as well as the number of predictors.

### Interpretation of the final model

In terms of selection, it is not given which explanatory variables are prioritized in *n* << *p* type datasets as this may be strongly dependent on properties of the dataset, in particular the correlations between the predictors. Moreover, it is not clear whether the selected predictors are the ones with the strongest association with the outcome [5]. Due to the penalty term described above (see the elastic net equation), the explanatory variables selected are influenced by every other variable selected. Biasing coefficient estimates by changing the penalty parameter *λ* or training elastic net models with datasets having specific properties may therefore lead to unpredicted results [20] and the selected variables could have no true relation with the outcome but be correlated with other predictor variables. Hence, as the elastic net does not have “oracle properties” [21, 22], it is not guaranteed that the selected set of variables is correct or truly related to the outcome [20]. In EWAS studies, this may be problematic as the chosen regression model may indirectly select CpGs associated with irrelevant genes or regions. The adaptive Lasso attempts to remedy the short-coming of unpredictable variable selection by providing oracle-like features [21]. However, the adaptive Lasso also has problems with collinearity [13]. An alternative method is the adaptive elastic net, which handles collinearity like the elastic net and has the oracle property like adaptive Lasso [23].

## Additional file

Additional file 1: Walk-through for glmnet predictions. (R 1 kb)

## Data Availability

The dataset described in the guidelines can be downloaded from the GEO database accession numbers GSE41169 (training dataset) and GSE36064 (prediction dataset).

## Abbreviations

EWAS: Epigenome-wide association studies
MSE: Mean squared error
SE: Standard error
